# Synergistic Biocontrol of *Agrobacterium tumefaciens* by Phage PAT1 and Ascaphin-8: Enhanced Antimicrobial Activity and Virulence Attenuation via HupB Loss

**DOI:** 10.3390/ijms26199355

**Published:** 2025-09-25

**Authors:** Miloud Sabri, Kaoutar El Handi, Cosima Damiana Calvano, Mariachiara Bianco, Angelo De Stradis, Toufic Elbeaino

**Affiliations:** 1 International Centre for Advanced Mediterranean Agronomic Studies (CIHEAM of Bari), Via Ceglie 9, 70010 Valenzano, Italy; miloud.sabri@uit.ac.ma (M.S.); elhandi@iamb.it (K.E.H.); 2 Interdepartmental SMART Center, Department of Chemistry, University of Bari, Via E. Orabona 4, 70126 Bari, Italy; mariachiara.bianco@uniba.it; 3 National Research Council of Italy (CNR), Institute for Sustainable Plant Protection (IPSP), University of Bari, Via Amendola 165/A, 70126 Bari, Italy; angelo.destradis@cnr.it; 4 National Research Council of Italy (CNR), Institute for Sustainable Plant Protection (IPSP), Piazzale Enrico Fermi, 1, 80055 Portici, Italy

**Keywords:** phage resistance, fitness trade-offs, phage-peptide synergy, proteomics, crown gall disease

## Abstract

*Agrobacterium tumefaciens* (*A. tumefaciens*), the causal agent of crown gall disease on several plant species, is responsible for substantial yield losses worldwide. The limitations of conventional pesticides in controlling this disease highlight the need for alternative antibacterial solutions. Phage biocontrol can be an option, effectively managing bacterial plant diseases, by reducing pathogen loads while driving evolutionary trade-offs, often enhancing synergy with other antibacterial strategies. In this study, we aimed to explore and develop a sustainable strategy to control *A. tumefaciens*, by combining *Agrobacterium* phage PAT1 with the natural antimicrobial peptide “Ascaphin 8” and leveraging the fitness trade-offs resulting from phage resistance. In vitro and in planta investigations showed that PAT1 in combination with Ascaphin 8 at the sublethal concentration of 3 μM could effectively eradicate *A. tumefaciens* in YPG broth and reduce tumor formation by 46.33% on tomato plants, unlike their individual applications, indicating that the combination was synergistic against *A. tumefaciens*. This synergy was attributed to the fitness trade-offs in *A. tumefaciens* induced by phage resistance, which led to increased sensitivity to antimicrobial peptides, slower growth rate, and an 89.96% attenuation of virulence in the PAT1-resistant mutant (AT-M1). Transmission electron microscopy analyses showed that treatment with 1 µM of Ascaphin 8 induced cytoplasmic condensation in 80% of AT-M1 cells, whereas only 16% of the wild-type CFBP 5770 cells exhibited similar alterations under identical conditions. Furthermore, proteomic analyses performed on AT-M1 and CFBP 5770 revealed that the mutant AT-M1 exhibited a loss of DNA-binding protein HupB and downregulation of SDR family oxidoreductase and superoxide dismutase. These molecular alterations are potentially associated with the reduced virulence and heightened AT-M1 sensitivity. This study investigated the fitness costs associated with phage resistance in *A. tumefaciens* and laid the first foundation for potential biocontrol of plant bacterial diseases, particularly *A. tumefaciens* infections, using phage–peptide combination.

## 1. Introduction

*Agrobacterium tumefaciens* (*A. tumefaciens*) is a globally significant bacterial plant pathogen responsible for crown gall disease in nearly 600 plant species, including many economically important plants such as apple, pear, cherry, apricot, almond, and walnut [1,2]. Crown gall leads to significant economic losses, particularly in orchards and nurseries, by reducing tree productivity, rendering nursery stock unsellable, and increasing susceptibility to environmental stress (e.g., drought and nutrient deficiencies) and opportunistic pathogens [3]. Through genetic transformation of plant cells, primarily mediated by the virulence (vir) gene cluster located on the Ti plasmid, *A. tumefaciens* induces uncontrolled cell proliferation, resulting in gall formation on roots and the crown, which disrupts nutrient uptake and causes irreversible crop damage [4]. In the United States, annual losses attributable to crown gall are estimated to exceed $20 million [5]. In China, infection rates can be as high as 30–100%, with production losses around 30%, and the incidence rate is reported to be increasing year by year [5]. The interactions between *A*. *tumefaciens* and host plants have been extensively studied due to its natural property as an efficient DNA delivery vector, leading to advancements in plant biotechnology. Nevertheless, limited progress has been achieved in the control of *A. tumefaciens*, and it remains a serious threat to agriculture [6]. Similar to other bacterial diseases, crown gall is difficult to control with conventional chemical-based bactericides [3]. Therefore, the development of sustainable and effective antibacterial tools against *A. tumefaciens* is of paramount importance.

Virulent (lytic) bacteriophages, which are viruses that specifically infect and lyse bacteria, are certainly among the most promising options in the field of biological control. Lytic bacteriophages are ubiquitous in the environment, recognized as safe agents, and their role as potent antibacterial agents in agriculture has been well documented [7,8,9]. The use of bacteriophages for biocontrol presents both benefits and limitations. Among the most significant advantages are their high host specificity and the unique ability to proliferate after application by targeting and eliminating bacterial hosts, unlike conventional antimicrobial compounds that tend to degrade over time [10]. However, their specific ability to kill bacteria does not lead to the total disappearance of susceptible host populations in an ecological niche, because bacteria can rapidly develop phage resistance during treatment [11,12]. Phage resistance is one of the major obstacles in developing effective phage therapies against bacteria, even though it is generally not as transferable to other microorganisms as antibiotic resistance markers [9,13]. Bacteria can resist phage attack through different mechanisms, including receptor modification to block phage attachment, the adaptive immunity of CRISPR-Cas, restriction-modification (RM) systems that degrade foreign phage DNA, and abortive infection (Abi) systems that trigger self-destruction to limit phage replication and spread [14]. Nevertheless, bacteria pay a cost for phage resistance, resulting in altered fitness and/or reduced virulence [15]. Moreover, it is noteworthy to mention that not all bacteria that have developed phage resistance suffer such costs. It depends on the mechanisms by which phage resistance is conferred, as well as the environmental context of the bacterium [12]. Unfortunately, there is limited knowledge about such costs paid by phage-resistant plant pathogenic bacteria.

To wisely leverage the costs incurred by phage-resistant pathogenic bacteria, the use of another antibacterial agent in combination with phages can strategically exploit the altered bacterial fitness for enhanced disease control. Among such attractive antibacterial compounds in the pipeline are natural antimicrobial peptides (AMPs), which are self-defense mechanisms of organisms, present in different lifeforms in nature [16]. AMPs can inhibit or kill a broad spectrum of microorganisms, including bacteria, often by non-specific mechanisms; hence, the acquisition of resistance to these antimicrobials in bacteria is much slower or rare [17,18]. However, the wider therapeutic application of AMPs is also hindered by their high synthesis cost and potential toxicity at high concentrations [19]. To cope with these shortcomings, it is essential to foster the antimicrobial effect of AMPs, enabling their effectiveness at significantly lower concentrations. Recent studies have highlighted the synergistic potential of combining phage therapy with AMPs to enhance bacterial suppression while reducing AMP biotoxicity and synthesis costs [20,21]. For this purpose, our study focuses on controlling crown gall disease using a cost-effective and eco-friendly strategy, by combining phage PAT1—a recently discovered bacteriophage shown to exhibit significant lytic activity against *A. tumefaciens* [22]—with the frog skin AMP “Ascaphin 8” at minimal concentrations. Therefore, the potential of PAT1 to enhance the antibacterial potency of Ascaphin 8 against *A. tumefaciens* and the fitness costs incurred by PAT1-resistant mutants of *A. tumefaciens* both in vitro and in planta were investigated. In addition, the impact of PAT1 resistance on *A. tumefaciens* at the proteomic level was explored using soft ionization techniques, including matrix-assisted laser desorption ionization (MALDI) and electrospray ionization (ESI), coupled with mass spectrometry (MS), to identify the proteins potentially linked to reduced virulence and heightened sensitivity of the phage-resistant mutant to AMPs.

## 2. Results

### 2.1. Sublethal Concentration of Ascaphin 8 Against A. tumefaciens

**Figure 1:**
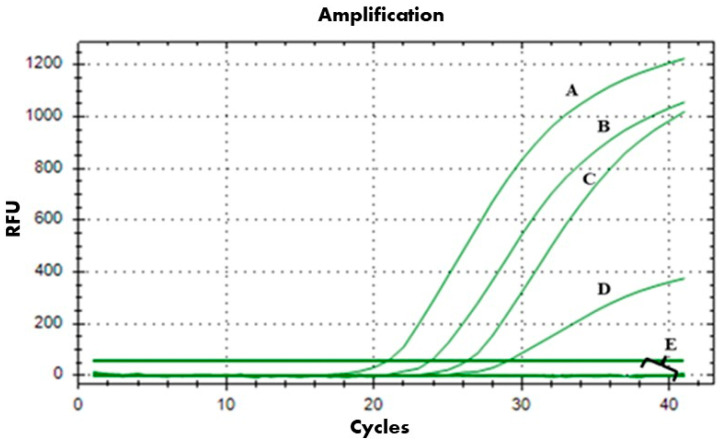
Viability-qPCR assay showing DNA amplification curves obtained from: (A): untreated *A. tumefaciens* (OD600 = 0.2); (B) Ascaphin 8-treated *A. tumefaciens* at 1 µM, (C) Ascaphin 8-treated *A. tumefaciens* at 2 µM; (D) Ascaphin 8-treated *A. tumefaciens* at 3 µM; (E) Ascaphin 8-treated *A. tumefaciens* at 5 and 8 µM and sterile water used as a negative control.

The spot assay results showed that, among the four AMPs tested, Ascaphin 8 exhibited the strongest antibacterial activity against *A. tumefaciens* on YPGA agar plates (Supplementary Figure S2). Thus, the bactericidal activity of Ascaphin 8 against *A. tumefaciens* was further evaluated at a range of low concentrations (8, 5, 3, 2, and 1 µM) using the viability-qPCR assay. The viability-qPCR of *A. tumefaciens* treated with Ascaphin 8 at 8 µM and 5 µM showed no amplifications, indicating complete bacterial lysis after 3 h of contact (Figure 1). Conversely, DNA amplifications were observed at 3, 2, and 1 µM in a concentration-dependent manner (Figure 1), indicating their non-lethal effects. Thus, 3 μM was selected as the sublethal concentration of Ascaphin 8 for further investigation in this study.

### 2.2. Synergistic Antibacterial Activity of PAT1-Ascaphin 8 Combination Against A. tumefaciens

**Figure 2:**
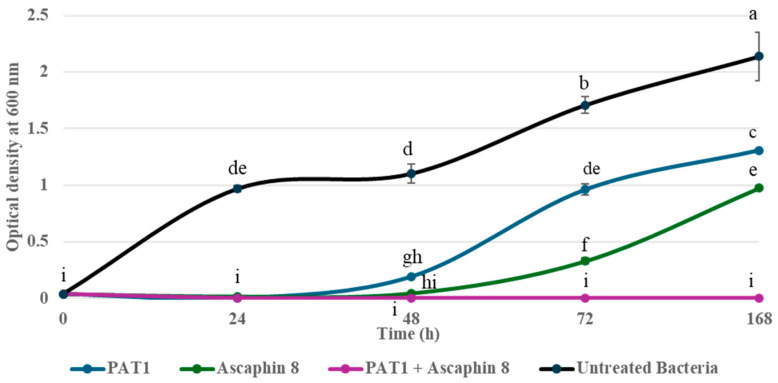
In vitro killing curves displaying the antibacterial activity of PAT1 and Ascaphin 8 separately and together against *A. tumefaciens* growth. The optical density of treated and untreated bacterial cultures at a 24 h interval for 7 days is compared. Data represent the mean ± standard deviation of three biological replicates (*n* = 3). Different letters (a–i) above the growth curves indicate statistically significant differences (*p* < 0.05) between treatments.

**Figure 3:**
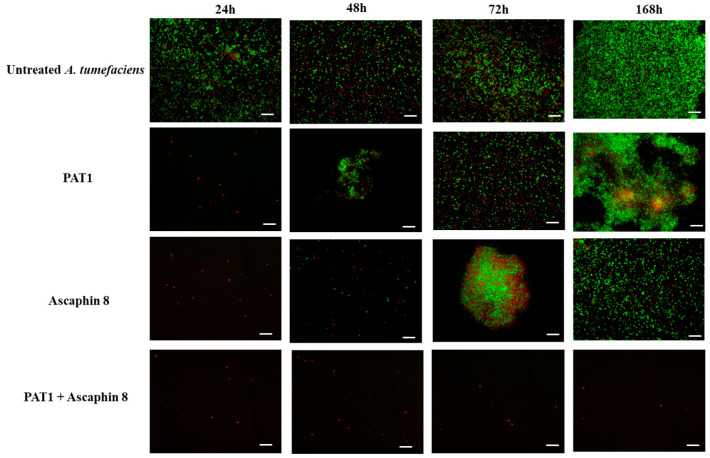
Fluorescent micrographs showing the bactericidal activity of PAT1 and Ascaphin 8 separately and in combination against *A. tumefaciens*. Green and red fluorescence represent live and dead cells, respectively. Bar: 10 µm.

**Figure 4:**
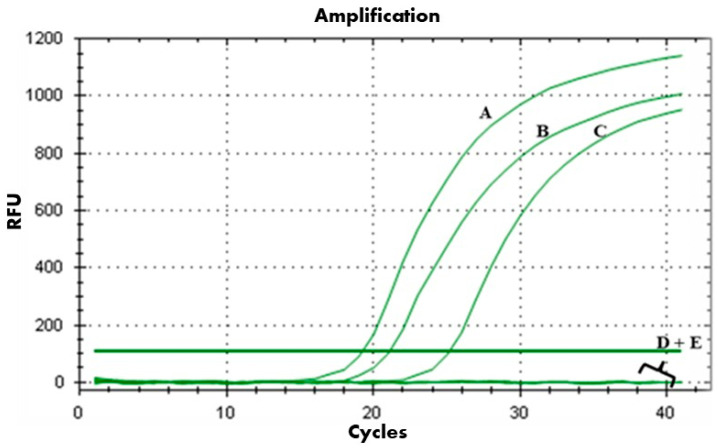
Viability-qPCR assay showing DNA amplification curves obtained from: (A) untreated *A. tumefaciens*; (B) PAT1-treated *A. tumefaciens*; (C) Ascaphin 8-treated *A. tumefaciens*; (D) PAT1 + Ascaphin 8-treated *A. tumefaciens*; all at 7 days of incubation. (E): sterile water used as a negative control. The 7-dpi time point for viability-qPCR was selected based on the progression shown in Figure 3, where treatment-specific differences in bacterial colonization became more stable and pronounced.

The in vitro antibacterial effect of PAT1 (at 10^8^ PFU/mL) in combination with Ascaphin 8 (at 3 μM) against *A. tumefaciens* was assessed over a 7-day incubation period using bacterial reduction assay, FM, and viability-qPCR assays. The results of the bacterial reduction assay and FM analyses showed that when PAT1 and Ascaphin 8 were applied separately against *A. tumefaciens*, both exhibited strong inhibition and reduction in bacterial culture within the first 24 h of incubation, achieving inhibition rates of 99% and 92%, respectively (Figure 2). However, after 24 h, PAT1-treated and Ascaphin 8-treated bacteria started to display an increase in green fluorescence (live bacterial cells) and in OD reading (Figure 2 and Figure 3), indicating the resumption of bacterial growth. This increase continued gradually until it reached an OD of 1.30 for PAT1-treated cells and 0.98 for Ascaphin 8-treated cells on day 7 (Figure 2); at that point, therefore, the efficacy of PAT1 and Ascaphin 8 was reduced to 38.96% and 54.17%, respectively. The decrease in the efficacy of PAT1 and Ascaphin 8 after 24 h may be attributed to the emergence of resistant mutants and/or depletion of peptide activity. Remarkably, when PAT1 and Ascaphin 8 were combined, a significant synergistic effect was recorded (Figure 2 and Figure 3). The results of the bacterial reduction assay showed that the sublethal concentration of Ascaphin 8, together with the PAT1 (at 10^8^ PFU/mL), resulted in a persisting inhibitory effect for 7 days, leading to the complete restriction of *A. tumefaciens* growth. The FM micrographs obtained corroborate the results of the bacterial reduction assay, confirming that PAT1-Ascaphin 8 combination exhibits significant and persistent destructive activity on *A. tumefaciens*, as evidenced by the red channels (lysed bacterial cells) and the absence of green color (Figure 3). Furthermore, the viability-qPCR assay conducted on genomic DNA extracted from untreated and treated bacteria after 7 days of incubation showed no DNA amplification in the curves of *A. tumefaciens* treated with PAT1 together with Ascaphin 8, unlike those of *A. tumefaciens* treated separately with PAT1 and Ascaphin 8 (Figure 4), confirming the complete eradication of *A. tumefaciens* when treated with PAT1 in combination with Ascaphin 8. These results showed that phage PAT1 in combination with the antimicrobial peptide Ascaphin 8 could effectively tackle *A. tumefaciens* infections.

### 2.3. AT-M1 and AT-M2 Susceptibility to PAT1 and Ascaphin 8

**Figure 5:**
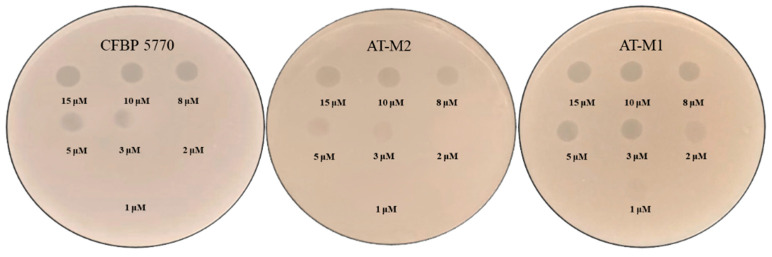
Spot assays showing the sensitivity of the wild-type strain CFBP 5770, AT-M1, and AT-M2 to different concentrations of Ascaphin 8.

To further investigate the cause of the lower efficacy observed with the individual applications of PAT1 and Ascaphin 8 against *A. tumefaciens*, a susceptibility test of isolated bacteria was conducted. The results showed that all colonies isolated from the Ascaphin 8-treated *A. tumefaciens* tube after 7 days of incubation, including the randomly selected isolate AT-M2, were susceptible to Ascaphin 8 in the same manner as the wild-type strain CFBP 5770 (Figure 5). This observation suggests that the decrease in the efficacy of Ascaphin 8, which began after 24 h of contact with *A. tumefaciens*, may be attributed to peptide depletion; however, this remains a hypothesis based on observed trends and has not been experimentally confirmed. Additionally, all colonies isolated from the PAT1-treated *A. tumefaciens* tube, including AT-M1 (randomly chosen for further investigation), were resistant to PAT1, confirming the emergence of resistance to PAT1. Remarkably, when the PAT1-resistant mutant AT-M1 was exposed to different concentrations of Ascaphin 8 (15, 10, 8, 5, 3, 2, and 1 μM), it demonstrated an increased sensitivity compared to the wild-type strain (Figure 5).

### 2.4. Comparative Sensitivity of Wild-Type Strain CFBP 5770 and AT-M1 Mutant to Different Antimicrobial Peptides

**Figure 6:**
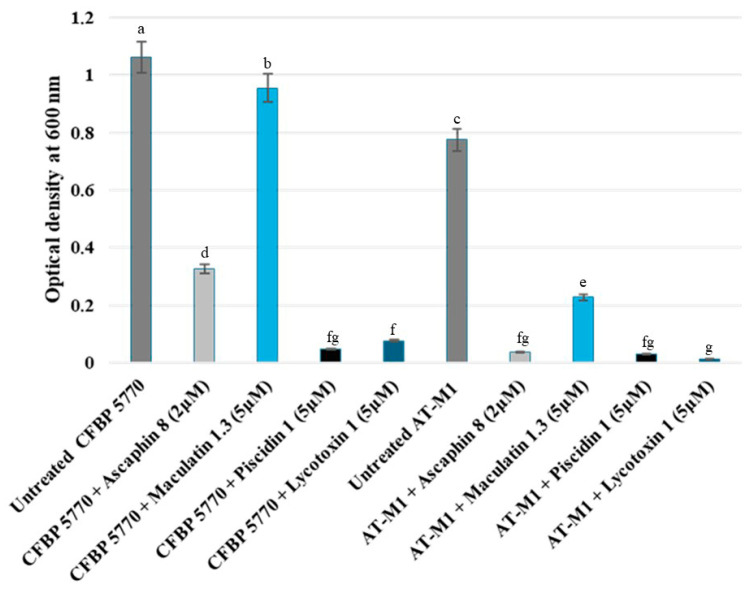
Histograms showing differential sensitivity of *A. tumefaciens* strain CFBP 5770 and PAT1-resistant mutant AT-M1 to antimicrobial peptides. The optical density of treated and untreated bacterial cultures with antimicrobial peptides after 24 h is compared. Data represent the mean ± standard deviation of three biological replicates (*n* = 3). Different letters (a–g) above the bars indicate statistically significant differences (*p* < 0.05) between treatments. The bar colors were selected to facilitate comparison between the wild-type and mutant strains in response to AMPs.

**Figure 7:**
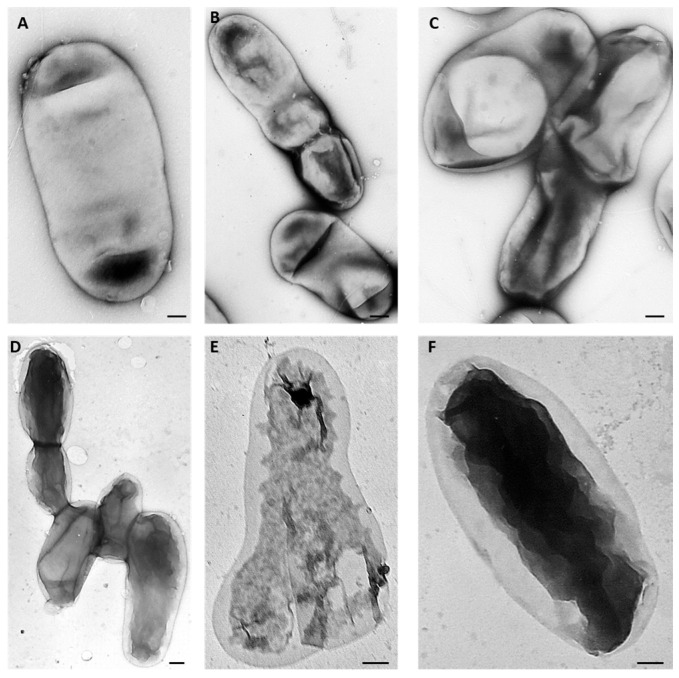
Transmission electron micrographs showing the impact of Ascaphin 8 at 1 μM on *A. tumefaciens* strain CFBP 5770 and PAT1-resistant mutant AT-M1. (**A**) Untreated *A. tumefaciens* cell used as a control. (**B**,**C**) Ascaphin 8-treated wild-type strain cells. (**D**–**F**) Ascaphin 8-treated AT-M1 cells showing cytoplasmic condensation. Bar: 500 nm.

The response of the AT-M1 mutant and the wild-type CFBP 5770 strain to various AMPs was assessed to determine whether resistance to PAT1 led to increased sensitivity to AMPs. The results of the turbidity test after 24 h of incubation in YPG broth medium showed that AT-M1 exhibited increased sensitivity to the tested AMPs compared to the wild-type strain CFBP 5770. At 2 µM, Ascaphin 8 inhibited the growth of AT-M1 and CFBP 5770 by 95% and 69%, respectively (Figure 6). At 5 µM, Lycotoxin I, Piscidin 1, and Maculatin 1.3 inhibited AT-M1 growth by approximately 98%, 96%, and 71%, respectively, while they inhibited the growth of CFBP 5770 by 92%, 95%, and 10%, respectively (Figure 6). Additionally, there was a 27% decrease in the growth rate of untreated AT-M1 compared to untreated CFBP 5770 (Figure 6). Moreover, TEM results revealed that only 16% of wild-type strain CFBP 5770 cells treated with Ascaphin 8 at 1 µM showed alterations (i.e., cytoplasmic condensation). However, when the mutant AT-M1 was treated with Ascaphin 8 at 1 µM, 80% of cells presented cytoplasmic condensation (Figure 7), confirming AT-M1’s higher sensitivity to Ascaphin 8. These results demonstrated that *A. tumefaciens*, which became more sensitive to AMPs with a reduced growth rate, pays a fitness cost for resistance to PAT1.

### 2.5. Impact of PAT1 Resistance on the Virulence of A. tumefaciens

**Figure 8:**
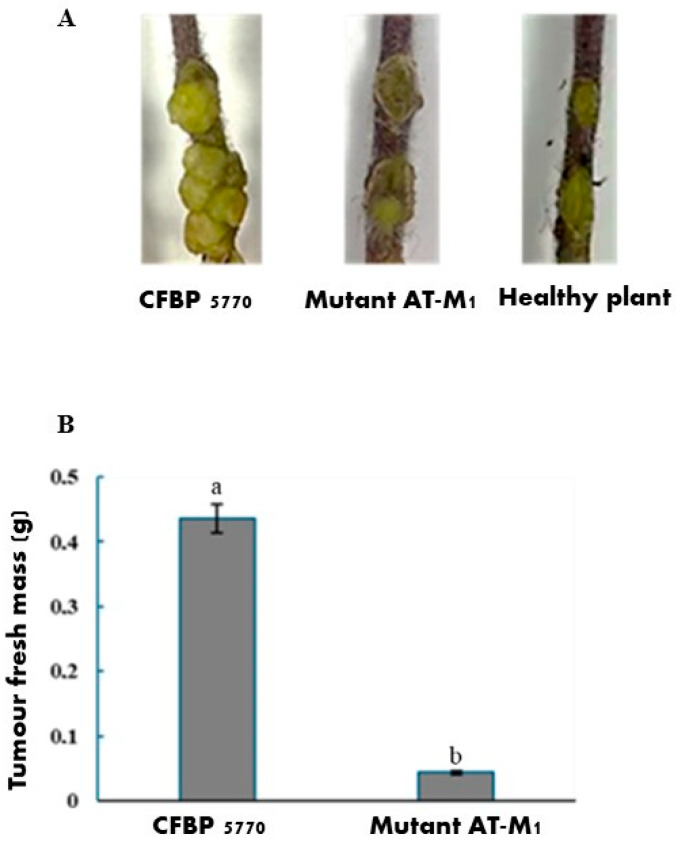
Virulence test showing the impact of PAT1 resistance on the virulence of *A. tumefaciens* in tomato plants. (**A**): Representative 45-day-old crown gall tumors on tomato stem induced by *A. tumefaciens* CFBP 5770 and the PAT1-resistant mutant AT-M1. (**B**): The mean fresh mass of tumors induced by CFBP 5770 and mutant AT-M1 (*n* = 10). Data represent the mean ± standard deviation of ten biological replicates (*n* = 10). Different letters (a and b) above the bars indicate statistically significant differences (*p* < 0.05) between treatments.

To assess the impact of PAT1 resistance on the virulence of *A. tumefaciens*, an in planta evaluation was performed on tomato plants. The virulence of the PAT1-resistant mutant and the wild-type strain was estimated by calculating variations in tumor formation based on the fresh mass of the tumors formed after 45 days post-inoculation (dpi). The results revealed that the virulence potency of the AT-M1 mutant was significantly lower than that of CFBP 5770, showing a decrease of 89.96% (Figure 8). These findings showed that PAT1 resistance is also related to lower virulence, leading to a significant attenuation in the tumor-inducing capacity of PAT1-resistant mutants. This reduction in virulence, coupled with fitness trade-offs resulting from PAT1 resistance, potentiates the synergistic antibacterial role of PAT1 in controlling *A. tumefaciens*.

### 2.6. The In Planta Antibacterial Efficacy of PAT1, Ascaphin 8, and Their Combination Against A. tumefaciens

**Figure 9:**
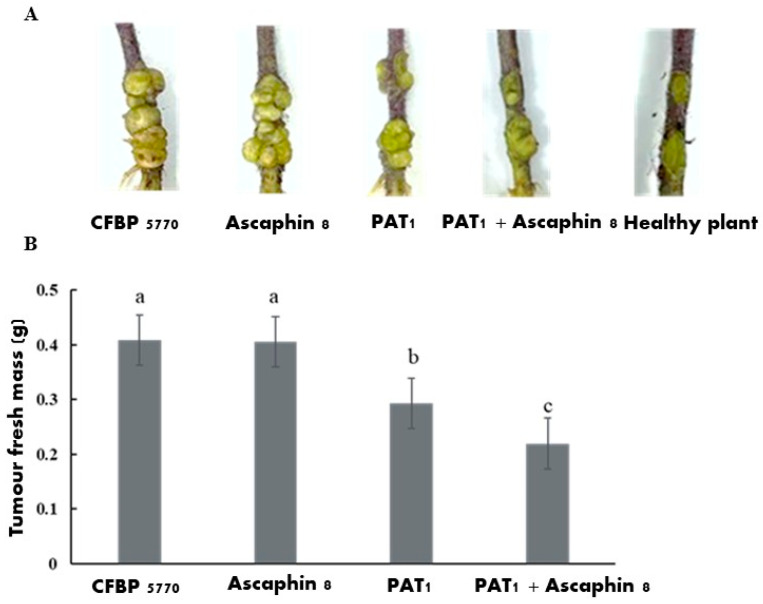
In planta assay showing the efficacy of PAT1 and Ascaphin 8, both individually and in combination, in controlling crown gall disease induced by *A. tumefaciens* CFBP 5770. (**A**): Representative 45-day-old crown gall tumors on tomato stems induced by different treatments, as indicated. (**B**): The mean fresh mass of tumors induced by PAT1 and Ascaphin 8-treated and untreated CFBP 5770 (*n* = 10). Data represents the mean ± standard deviation of ten biological replicates (*n* = 10). Different letters (a–c) above the bars indicate statistically significant differences (*p* < 0.05) between treatments.

The antibacterial effects of PAT1 and Ascaphin 8, both individually and in combination, against *A. tumefaciens* infections were assessed in tomato stems. The efficacy of each treatment in reducing *A. tumefaciens* symptoms was estimated by calculating variations in tumor formation based on the fresh mass of the tumors formed 45 dpi. The analysis of our data revealed a very significant difference between the treatments (*p* = 0.000) (Figure 9), and results showed that all untreated *A. tumefaciens*-infected plants (positive controls) displayed significant tumor formation, and no tumors were observed in healthy plants (Figure 9). Applying Ascaphin 8 alone at its sublethal concentration of 3 µM resulted in a tumor formation level comparable to that of the positive control, indicating that at this concentration, Ascaphin 8 was ineffective in controlling *A. tumefaciens* within the plants (Figure 9). Conversely, PAT1 alone, which was less effective in vitro than Ascaphin 8, reduced tumor formation by 28.19% compared to the positive control (Figure 9). Consistent with the in vitro results, the combined application of PAT1 and Ascaphin 8 resulted in a synergistic antibacterial effect against *A. tumefaciens* within tomato stems, reducing tumor formation by 46.33% (Figure 9). This finding corroborates the in vitro results, confirming the synergistic interaction between Ascaphin 8 and PAT1 and highlights the potential of PAT1-Ascaphin 8 combination in controlling *A. tumefaciens* infection in plants. At the end of the experiment, 15 *A. tumefaciens* bacteria, confirmed by PCR analyses, were reisolated from PAT1-treated tomato plants and 18 from PAT1 + Ascaphin 8-treated tomato plants. The results of the spot assay showed that none of these isolates were resistant to PAT1, suggesting that the plant environment may pose additional challenges that impede the survival and persistence of PAT1-resistant mutants.

### 2.7. Protein Identification in Wild-Type Strain and AT-M1 Mutant by Mass Spectrometry

**Figure 10:**
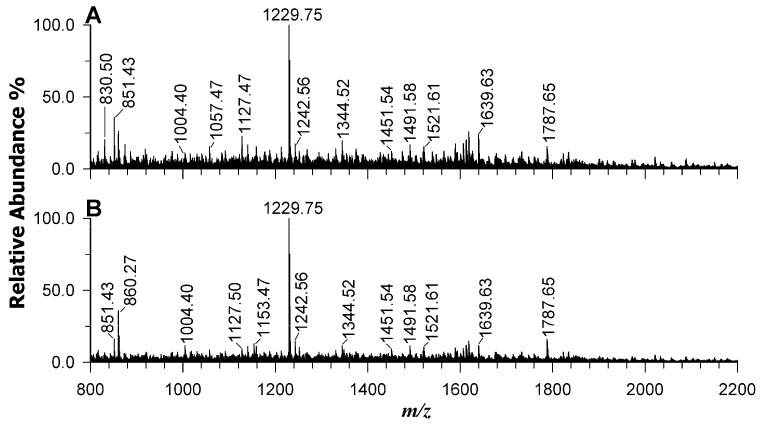
Peptide Mass Fingerprinting of tryptic digests obtained on Microcon filters for the (**A**) wild type strain and (**B**) mutant AT-M1 bacteria.

MALDI-MS analyses of protein digests of wild-type and mutant AT-M1 are reported in plot A and B of Figure 10, respectively. The PMF of the two bacteria shows very small differences concerning peptide’s intensities, being the ions at *m*/*z* 830.50, 851.43, 1057.47 and 1127.47 higher in wild type strain (A) with a fold change WT/AT-M1, respectively, of 2.7, 1.9, 1.6, 2.1 and the ions at *m*/*z* 860.27, 1153.47 and 1787.65 greater in AT-M1 sample with a fold change WT/AT-M1, respectively, of 0.6, 0.5, 0.8.

By processing these data using ProteinProspector MS-Fit tool, several proteins such as DNA ligase, malate synthase G, cysteine-tRNA ligase, and transaldolase, related to DNA replication and repair of damaged DNA, carbohydrate metabolism, catalytic activity, balance of metabolites were identified with high scores in both samples. The proteins retrieved from PMF, by protein name, and occurrence in wild type or/and mutant down to 10% of protein coverage are summarized in the Supplementary Table S1. Potentially, it was possible to highlight small differences in the incidence and scores of proteins, and these discrepancies were ascribed to variable suppression ion effects experienced by the complexity of samples analyzed in one spot by MALDI investigation. The recognized proteins were associated with a multispecies *Rhizobium*/*Agrobacterium* group, since it was not possible to restrict the search to *A. tumefaciens* in free access PMF. The distinction between the two bacteria was successful through a previous separation on SDS-PAGE and a further chromatographic separation of the in-gel tryptic digests.

The SDS-PAGE gel (Supplementary Figure S1), which allowed the separation of different molecular weight proteins, revealed the presence of four distinct bands in each strain. Specifically, band A (wild-type) and A′ (AT-M1) are in the 50–70 kDa range, bands B and B′ between 30 and 50 kDa, bands C and C′ in the 20–25 kDa range, and bands D and D′ between 10 and 15 kDa. Notably, the lower portion of band C and the entirety of band D appeared significantly more intense in the wild-type strain, while they were barely detectable in the AT-M1 mutant.

Table 1 reports the list of main protein families retrieved, the recognized peptides, and the specific occurrence in the investigated band. A preliminary comparison between the proteins identified via the MALDI-MS approach (Table S1) and those identified through SDS-PAGE followed by RPLC-MS analysis (Table 1) reveals that several protein families were detected by both methods. Proteins belonging to the ATP synthase, elongation factor, and ribosomal protein families were identified using both techniques. These families were primarily found in bands A and B of both species, the most intense bands in the electrophoretic separation, reflecting a higher protein content. It is not surprising that these same proteins were also detected using a shotgun proteomics approach, such as the MALDI-MS method. In this type of analysis, the ionization of the most abundant species is favoured, explaining their detection across both methods.

**Figure 11:**
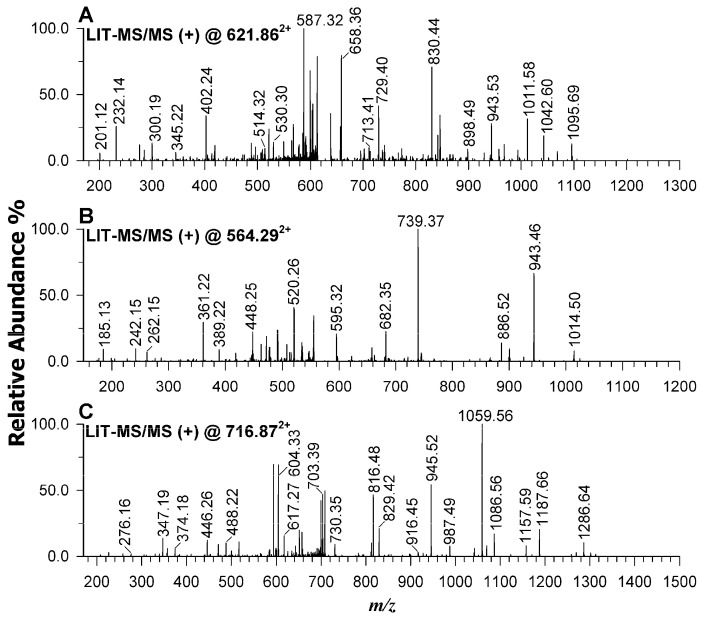
RPLC-ESI-MS/MS of peptides (**A**) TVVITAAGQGIGR at *m*/*z* 621.86^2+^ from SDR family oxidoreductase, (**B**) LAGFGSFSVSR at *m*/*z* 564.29^2+^, and (**C**) MNKNELVSAVAEK at *m*/*z* 716.87^2+^, both derived from DNA-binding protein HupB occurring only in wilt type samples.

In the low molecular weight bands (C and D), the SDR family oxidoreductase and superoxide dismutase proteins were detected with greater intensity in the wild-type sample, whereas the DNA-binding protein HupB was exclusively recovered in the wild-type strain. For illustration, Figure 11 presents the MS/MS spectra of the peptides TVVITAAGQGIGR (*m*/*z* 621.865^2+^), LAGFGSFSVSR (*m*/*z* 564.295^2+^), and MNKNELVSAVAEK (*m*/*z* 716.877^2+^), which are associated with the discriminant proteins.

## 3. Discussion

Phages are natural bacterial killers with dual action: directly lysing pathogens and indirectly inducing resistance, which often compromises bacterial fitness and virulence, increasing susceptibility to other antimicrobials and enabling potent combination therapies [15,23,24]. In this context, the natural AMP “Ascaphin 8” was tested against *A. tumefaciens*, demonstrating strong antibacterial activity at low concentrations, with 3 µM being the sublethal concentration. In terms of toxicity, Eley et al. (2008) [25] demonstrated that Ascaphin 8 is toxic to human erythrocytes, with an LC_50_ value of 55 μM when incubated with washed human erythrocytes (2 × 10^7^ cells) from a healthy donor in 100 μL of Dulbecco’s phosphate-buffered saline (pH 7.4) for 1 h at 37 °C. However, its high potency against *A. tumefaciens* at low concentrations potentiates its use in combination with PAT1, allowing for lower effective concentrations while reducing toxicity and synthesis costs. The combination of Ascaphin 8 at 3 µM with PAT1 (at 10^8^ PFU/mL) resulted in a sustained inhibitory effect against *A. tumefaciens* for 7 days, unlike treatments with PAT1 or Ascaphin 8 alone, leading to the complete eradication of *A. tumefaciens* cells. This synergistic effect suggests that the dual action of membrane disruption by Ascaphin 8 and phage-mediated lysis offers a promising strategy for controlling *A. tumefaciens* infections. Several studies have reported the potential of phages to act synergistically and enhance the activity of other antimicrobial agents, such as antibiotics and bacteriocins [26,27,28]. For example, Gu Liu et al. (2020) [29] concluded that lytic phages can resuscitate an ineffective antibiotic for previously resistant bacteria while also synergizing with antibiotics by lowering the antibiotic’s MIC. Furthermore, a combination of low-dose antibiotics and phages has been shown to effectively prevent the emergence of phage-resistant bacteria in a model of a urinary tract infection [30].

At the interaction level, the PAT1-resistant mutant AT-M1 exhibited enhanced sensitivity to lower concentrations of Ascaphin 8 compared to the wild-type strain. This increased sensitivity of AT-M1 was also observed with other AMPs tested (Maculatin 1.3, Piscidin 1, and Lycotoxin I), indicating the important role of PAT1 in increasing the effectiveness of AMPs against *A. tumefaciens*. These findings suggest that PAT1 infection induces genetic mutations in *A. tumefaciens* bacteria, creating conditions that lead to more positive treatment outcomes as a fitness trade-off for developing resistance. This may be due to phage-induced changes in bacterial membrane structure or physiology that compromise bacterial membrane barriers, thereby increasing sensitivity to AMPs. Furthermore, the AT-M1 mutant was found to be 89.96% less virulent on tomato plants than the wild-type strain, reaffirming PAT1’s considerable indirect impact on *A. tumefaciens* fitness. In this regard, the impact of PAT1 resistance on *A. tumefaciens* at the proteomic level was investigated, revealing that the AT-M1 mutant displayed a loss of DNA-binding protein HupB and downregulation of SDR family oxidoreductase and superoxide dismutase proteins. These three proteins play essential roles in various metabolic pathways, pathogenesis, gene regulation, and biofilm formation. They are critical for bacterial survival under host-mediated stresses and contribute to enhanced tolerance to key first-line antibiotics [31,32,33]. The loss or downregulation of these proteins in AT-M1 mutants can, thus, be associated with the increased sensitivity to AMPs and attenuated virulence. Indeed, Singh et al. (2022) [33] demonstrated that the loss of the HupB protein in *Mycobacterium tuberculosis* significantly enhances the permeability of the bacterial cell wall by modulating the levels of several surface lipids, possibly influencing overall susceptibility to host-mediated stresses. Additionally, the loss of hupB downregulates the expression of efflux pumps, thereby making *Mycobacterium tuberculosis* more vulnerable to antibiotics such as isoniazid and rifampicin [33]. Furthermore, Conforte et al. (2019) [32] showed that the histone-like protein HupB plays an essential role in the pathogenesis of *Xanthomonas citri* ssp. *citri* through the regulation of biofilm formation and biosynthesis of the flagellum. Additionally, Saenkham et al. (2007) [31] demonstrated that superoxide dismutase plays a significant role in the virulence of *A. tumefaciens*, showing that the ability of a *sodBI* mutant of *A. tumefaciens* to induce tumors on tobacco leaf discs was significantly reduced. Moreover, SDR family oxidoreductase plays critical roles in lipid, amino acid, carbohydrate, cofactor, hormone, and xenobiotic metabolism as well as in redox sensor mechanisms, helping bacteria to adapt to various environmental conditions and host interactions [34]. In our study, considering the critical roles of DNA-binding protein HupB, SDR family oxidoreductase, and superoxide dismutase proteins in pathogenesis, adaptation, metabolism, and bacterial cell wall permeability, it is evident that the altered expression of these proteins in *A. tumefaciens* due to PAT1 resistance likely contributed to the attenuated virulence, slower growth rate, and increased sensitivity to AMPs. Although the underlying cause of phage resistance remains unclear, it is most associated with genetic mutations. However, the possible involvement of epigenetic regulation cannot be excluded and warrants further investigation in future studies. Nevertheless, PAT1 has been demonstrated to be a valuable tool for controlling *A. tumefaciens* infections within an integrated disease management approach. We acknowledge that direct experimental validation of the roles of these three proteins is beyond the scope of this study, and we emphasize the need for future functional analyses to confirm their specific contributions to the observed phenotypes. Furthermore, the absence of PAT1-resistant mutants in planta after 45 dpi likely reflects compromised survival due to altered protein expression.

The results of the in planta experiments were consistent with the in vitro findings, showing a synergistic antibacterial effect of PAT1-Ascaphin 8 combination against *A. tumefaciens* within tomato stems, resulting in a 46.33% reduction in tumor formation. PAT1 alone reduced the fresh tumor mass by 28.19%, whereas Ascaphin 8, at a low concentration of 3 μM, proved ineffective in controlling *A. tumefaciens* in planta. These findings suggest that PAT1 exerted dual effects in tomato plants by combating *A. tumefaciens* infections directly and synergistically enhancing the activity of Ascaphin 8 against *A. tumefaciens*. Additionally, the use of a higher concentration of Ascaphin 8 in combination with PAT1 may enhance the biocontrol efficacy.

## 4. Materials and Methods

### 4.1. Peptide Synthesis, Bacteriophage, and Bacterial Growth Conditions

The peptides listed in Table 2 were selected based on their reported bactericidal efficacy in the literature against various plant pathogenic bacteria, such as *Xylella fastidiosa* and *Erwinia amylovora*, primarily through disruption of bacterial membrane integrity [35,36]. 

They were synthesized by ProteoGenix (Schiltigheim, France) using standard Fmoc solid-phase peptide synthesis (SPPS), followed by purification via reverse-phase HPLC on a C18 column using a 5–55% acetonitrile gradient with 0.1% trifluoroacetic acid (TFA). The purified peptides were confirmed to be ≥95% pure by analytical HPLC and their identity verified by electrospray ionization mass spectrometry (ESI-MS). Lyophilized peptides were stored at −20 °C. For use, peptides were dissolved in sterile Milli-Q water to a stock concentration of 1 mM and filter sterilized through a 0.22-μm nylon Acrodisc^®^ syringe filter (Merck, Rome, Italy). Stability was confirmed for at least six months by periodic HPLC and MS analysis to ensure reproducibility across experiments.

*Agrobacterium tumefaciens* strain CFBP 5770 was grown either at 28 °C in liquid yeast extract peptone glucose broth (YPG) (5.0 g/L yeast extract, 5.0 g/L peptone, and 10.0 g/L glucose) or on yeast extract peptone glucose agar (YPGA, i.e., YPG supplemented with 1.5% agar). The strain was stored at −80 °C in 25% glycerol in YPG broth. Before use, the strain was plated from glycerol stocks onto YPGA agar plates and incubated at 28 °C for 24 h. The bacteriophage *Agrobacterium* phage PAT1 (GenBank accession number: PQ082932) used in this study was isolated from sewage water via enrichment culture using the host strain CFBP 5770 grown in YPG broth at 28 °C. Phage purification was performed through three successive rounds of plaque picking using the double agar overlay method, and the phage titer was determined by a double-layer agar assay [38]. Key characteristics of PAT1, including its host range, morphology, genomic features, and biological properties such as stability across varying pH levels and temperatures, have been previously reported [22].

### 4.2. Bactericidal Activity of Ascaphin 8 Against A. tumefaciens

The four AMPs listed in Table 2 were initially screened using the spot-on-lawn method [39] to select the peptide exhibiting the strongest antibacterial activity against *A. tumefaciens*, based on the concentration-effect relationship (activity at lower concentrations) and the clarity of inhibition observed on the bacterial lawn. Accordingly, the bactericidal activity of Ascaphin 8 was further assessed by a contact test, coupled with viability-quantitative PCR (viability-qPCR) using the PMAxx™ (Biotium, Rome, Italy) [40]. Briefly, 50 µL of *A. tumefaciens* strain CFBP 5770 suspensions at OD600 of 0.2 were incubated with 50 µL of Ascaphin 8 at 5, 3, 2, and 1 μM for 3 h at 28 °C. After incubation, samples were treated with PMAxx™ at a final concentration of 7.5 μM, incubated in the dark at room temperature for 8 min, and followed by a 15 min photoactivation step using the PMA-Lite™ LED Photolysis Device (Biotium, Rome, Italy). Genomic DNA of all samples was extracted using the DNeasy Plant Extraction kit (Qiagen, Milan, Italy). Viability-qPCRs were carried out in a thermocycler apparatus (Bio-Rad CFX96, BioRad, Milan, Italy), using the primer pair virD2A/virD2C and conditions reported in Ganjeh et al. (2020) [41]. The viability-qPCR cycles included an initial denaturation step at 95 °C for 10 min, followed by 40 cycles of 95 °C for 30 s, 60 °C for 40 s, and 72 °C for 30 s, with fluorescence readings at each cycle.

### 4.3. In Vitro Antibacterial Potency of PAT1 and Ascaphin 8 Combination Against A. tumefaciens

The combination of PAT1 and Ascaphin 8 can result in (i) additive effects, in which their combined efficacy is equal to the sum of their individual effects, (ii) synergistic effects, with significantly greater combined efficacy, or (iii) antagonistic effects, with the molecular action of one agent interfering with the other. Before conducting experiments using the PAT1-Ascaphin 8 combination, the potential impact of Ascaphin 8 on PAT1 was assessed by treating the phage (10^8^ PFU/mL) with Ascaphin 8 (3 μM) for 24 h at room temperature. Subsequently, serial dilutions were performed with phage buffer to minimize the concentration of the peptide to a very low level, and the phage titer was determined using the double agar overlay method and compared with the untreated PAT1.

To explore the PAT1-Ascaphin 8 combination, the growth kinetics of *A. tumefaciens* strain CFBP 5770 were assessed in the presence of PAT1 (10^8^ PFU/mL), Ascaphin 8 (3 μM), and the combination of PAT1 and Ascaphin 8. Experiments were conducted in 1.5 mL Eppendorf tubes with a final volume of 975 µL, comprising 900 µL of YPG medium, 25 µL of CFBP 5770 (OD_600_ = 0.2), and 25 µL of each agent, and the tubes were incubated at 28 °C for 7 days. Optical density measurements were taken at four time points (24 h, 48 h, 72 h, and 7 days) using the NanoDrop™ One/OneC Microvolume UV-Vis Spectrophotometer (Thermo Fisher, Waltham, MA, USA), while photomicrographs were acquired at the same time points using Fluorescence Microscopy (FM) [42]. After 7 days of incubation, a viability-qPCR was conducted to validate and confirm the obtained results. Additionally, bacteria that grew in the tubes containing PAT1 and Ascaphin 8 separately were transferred to YPG plates, from which two isolates were randomly selected for further studies and assigned as AT-M1 and AT-M2, respectively.

### 4.4. Susceptibility Test of AT-M1 and AT-M2 to PAT1 and Ascaphin 8

To evaluate whether the two isolates AT-M1 and AT-M2 had developed resistance to PAT1 and Ascaphin 8, respectively, drops of 10 μL of PAT1 solution (10^8^ PFU/mL) and Ascaphin 8 at 15, 10, 8, 5, 3, 2, and 1 μM were spotted onto bacterial lawns using the spot assay as described above. CFBP 5770 was utilized as a control to compare the susceptibility of the two mutants with the wild-type strain.

### 4.5. Antimicrobial Peptide Sensitivity of the AT-M1 Mutant

To explore whether the phage-resistant *A. tumefaciens* mutant AT-M1 exhibited increased sensitivity to AMP activities, we conducted a turbidity assay using four natural peptides (Ascaphin 8 at 2 μM, Lycotoxin I at 5 μM, Maculatin 1.3 at 5 μM, and Piscidin 1 at 5 μM). In brief, 1.5 mL Eppendorf tubes containing 200 µL YPG broth medium were inoculated with 25 µL of bacterial suspension (OD_600_ = 0.2), and 25 µL of peptides were introduced. The tubes were then incubated at 28 ◦C for 24 h, and the optical density was measured (at 24 h) at OD600 using the NanoDrop™ One/OneC Microvolume UV-Vis Spectrophotometer. In addition, a transmission electron microscopy (TEM) assay was performed to scrutinize the differential sensitivity of the wild-type strain and mutant AT-M1 to Ascaphin 8 at 1 μM using the dip method [22]. Subsequently, ten different areas of specimen section from each sample were examined, and the percentage of altered bacterial cells was calculated.

### 4.6. In Planta Antibacterial Efficacy of PAT1 and Ascaphin 8 Against A. tumefaciens and Virulence Assessment of the AT-M1 Mutant in Tomato Plants

To assess the antibacterial potential of PAT1 and Ascaphin 8, both individually and in combination, in interfering with *A. tumefaciens* infection within plants, an in planta assay was conducted. Tomato plants (*Lycopersicum esculentum* cv. Ciliegia) were chosen for their susceptibility to *A. tumefaciens*, enabling reliable evaluation of the antibacterial activity of PAT1 and Ascaphin-8. Four treatments were performed: PAT1 (10^8^ PFU/mL), Ascaphin 8 at 3 μM, PAT1-Ascaphin 8 combination, and sterile water. Briefly, one-month-old tomato plants were wounded at two sites in the stem with a sterile scalpel. The wounds were inoculated with 50 µL of an overnight-enriched culture of CFBP 5770 (OD_600_ = 0.2), followed 30 min later by the addition of 50 µL of antibacterial agents. Plants were maintained at 25 °C in a greenhouse under a natural photoperiod and 60% relative humidity (RH). Each treatment consisted of 20 wounds, and the plants were visually inspected for gall development. After 45 days, the fresh mass of the formed galls was measured, and the percentage reduction in gall mass for each treatment was calculated using the following formula:

$$Disease\;reduction\left(\%\right)=100-(\frac{sum\;of\;tumor\;fresh\;mass\;(g)\;in\;treatment}{sum\;of\;tumor\;fresh\;mass\left(g\right)\;in\;positive\;control}\;\times 100)$$

To investigate the emergence of PAT1 resistance in tomato plants, galls formed at the end of the in planta experiment (45 dpi) were analyzed. Briefly, 1 g of formed galls from the PAT1 alone and PAT1 + Ascaphin 8 treatments were separately ground in 2 mL of sterile water and centrifuged at 1000× *g* for 5 min to remove plant debris. The resultant supernatant was diluted six times with a 10-fold serial dilution, and 50 μL were plated on YPGA plates. The plates were incubated at 28 °C for 48 h and *A. tumefaciens*-like colonies were purified on YPGA plates and subjected to PCR assays using the virD2A/virD2C primers as described earlier. The *A. tumefaciens* isolates, confirmed through PCR, were subsequently assessed for PAT1 resistance using a spot assay, as previously described.

Furthermore, to evaluate whether the virulence of phage-resistant mutant AT-M1 was attenuated, 50 µL of an overnight-enriched culture of AT-M1 (OD_600_ = 0.2) was introduced into tomato plants following the previously described method, and the results were compared with those obtained using the wild strain CFBP 5770. Each treatment consisted of 10 plants, and the virulence of the inoculated bacteria was assessed based on the fresh mass of the formed galls. All experiments in this study were conducted with experimental duplicates to ensure reliability.

### 4.7. Statistical Data Analysis

To test the biocontrol efficacy of PAT1 and Ascaphin 8, individually and in combination, against *A. tumefaciens* infections in tomato plants, a one-way analysis of variance (ANOVA) was performed using the IBM SPSS Statistics 26 software. The significance of differences was calculated by Duncan’s post hoc test, and a *p*-value less than 0.05 was considered statistically significant.

### 4.8. Proteomic Analysis of AT-M1 and Wild-Type Bacteria

#### 4.8.1. Protein Extraction and Purification

A volume of 300 µL of protein extraction buffer (7 M urea, 2 M thiourea, 2% CHAPS, 65 mM DTT, 40 mM Trizma base) (Merck KGaA, Darmstadt, Germany) was added to 35 mg of both wild-type and mutant (AT-M1) samples. Probe sonication (Hielscher Ultrasonics GmbH, Teltow, Germany) was performed to aid cell lysis and protein extraction, by running three cycles (ON/OFF) of 10 s with 5 Wh energy. The samples were shaken at 400 rpm for 2 h at 25 °C, and then centrifuged at 15,000× *g* at 20 °C for 15 min. The supernatant was collected and processed for protein purification by Microcon^®^ 10 kDa filter (Merck KGaA, Germany) [43] and/or for separation by SDS-PAGE. In detail, Microcon^®^ filters were activated by 100 µL of 0.1% formic acid (FA) and centrifuged at 12,000× *g* at 20 °C for 15 min. Then, 100 µL of protein extract were loaded and incubated at 56 °C, 300 rpm for 30 min, and then centrifuged. After washing with 100 µL of 50 mM Tris-HCl (pH 8.5), alkylation was performed by adding 100 µL of 40 mM IAA at 25 °C, 300 rpm for 30 min, and then centrifuged. Filters were washed twice with 50 mM ammonium bicarbonate (ABC), and protein digestion was accomplished on the filter by adding 0.02 µg/µL trypsin and leaving overnight under stirring at 300 rpm and 37 °C. After centrifugation, the peptide solution was collected, and the digestion was stopped by adding FA.

#### 4.8.2. SDS-PAGE and In-Gel Digestion

For SDS-PAGE analysis, protein quantification was performed using the Micro BCA Protein Assay Kit. Initially, proteins were precipitated using a cold acetone protocol, in which 400 µL of cold acetone was added to 200 µL of the protein extract. Samples were incubated at −20 °C for 30 min, followed by centrifugation at 20,000 rcf for 10 min. After centrifugation, the supernatant was discarded, and the resulting pellets were dried under nitrogen and then reconstituted in 200 µL of water and diluted appropriately for quantification. For SDS-PAGE separation, 7.5 µL of the 1:5 diluted protein extract (corresponding to 30 µg of protein) was mixed with 22.5 µL of Laemmli buffer containing 100 mM DL-dithiothreitol (DTT), and the mixture was heated at 100 °C for 5 min before loading onto the gel. Subsequently, the samples were loaded on precast Any kD^TM^ Mini-PROTEAN^®^ TGX™ gel and separation was carried out at 200 V for 20 min in running buffer 10× Tris/Glycine/SDS (25 mM Tris, 192 mM glycine, 0.1% SDS, pH 8.3) with Precision Plus Protein™ All Blue Prestained Protein Standards (10–250 kDa) as a marker (Bio-Rad Laboratories, Hercules, CA, USA). To visualize proteins, the gel was left in the staining solution of Coomassie Brilliant Blue R-250 dye (Gallagher, 2012) [44]. The bands of interest (A–D in wild-type and A′–D′ in mutant, see Supplementary Figure S1) were cut and, after washing, the in-gel digestion was performed by adding 100 µL of trypsin (0.02 µg/µL in H_2_O: acetonitrile (ACN) 91:9 *v*/*v*) at 37 °C and 400 rpm overnight. Then, the solution was collected and acidified before proceeding with MALDI and reversed-phase liquid chromatography (RPLC)-ESI-MS analyses.

#### 4.8.3. MALDI-MS and RPLC-ESI-MS of Tryptic Digests

Samples for MALDI-MS analyses were performed in triplicate and prepared by blending the tryptic digests with an equal volume of 4-chloro-α-cyano cynnamic acid (CClCA) matrix (10 mg/mL in ACN 70% with 0.1% of formic acid) (Merck KGaA, Germany) and depositing 1 µL of resultant mix on the target plate [45]. The employed instrument was a 5800 MALDI-ToF/ToF (SCIEX, Darmstadt, Germany) equipped with a neodymium-doped yttrium lithium fluoride (Nd:YLF) laser (345 nm), working in reflectron positive mode, in the range 800–4000 *m*/*z*, with a mass accuracy of 10 ppm. Typically, 1000 laser shots were accumulated by a random rastering pattern, at laser pulse rates of 400 Hz; an averaged mass spectrum was obtained by adding at least five single mass spectra (1000 laser shots each). The delayed extraction time was set at 250 ns. DataExplorer software 4.0 (Sciex, Framingham, MA, USA) was used to control the acquisitions and to perform the initial elaboration of data. The identification of proteins was attained by processing the peptide mass fingerprinting (PMF) spectra by ProteinProspector MS-Fit tool (Regents of the University of California, Oakland, CA, USA, v 6.6.4) with the following settings: *Agrobacterium* as taxonomy, trypsin as enzyme, max two missed cleavages, cysteine carbamidomethylation as constant modification, methionine oxidation as possible modification, and peptides tolerance 100 ppm.

RPLC-ESI-MS analyses, acquired in triplicate, were performed in positive mode on an Ultimate 3000 UHPLC chromatographic system coupled by a heated electrospray source ionization (HESI) with either a Velos Pro linear ion trap mass spectrometer or a Q-Exactive Orbitrap™ mass spectrometer (Thermo Scientific, Waltham, MA, USA). The chromatographic separations were carried out at 40 °C using a Phenomenex Aeris WIDEPORE 200 Å C18 column (250 × 2.1 mm, 3.6 μm) equipped with Phenomenex AJO 8783 WIDEPORE C18 (2 × 2.1 mm ID) security guard cartridge and a mobile phase composition based on water (solvent A) and ACN (solvent B), both containing 0.1% of FA [46]. The chromatographic gradient at a flow rate of 0.20 mL/min was the following: 0–2 min isocratic at 5% of solvent B; 2–90 min linear from 5% to 50% (*v*/*v*) of B; 90–95 min linear from 50% to 100% (*v*/*v*) of B; 95–100 min isocratic at 100% B; 100–105 min back to 5% (*v*/*v*) of B, followed by 5 min equilibration time. The ESI and ion optic parameters adopted for Orbitrap were the following: sheath gas flow rate, 60 (arbitrary units); auxiliary gas flow rate, 15 (arbitrary units); spray voltage, 4 kV in positive polarity; capillary temperature, 275 °C; S-lens radio frequency level, 100 arbitrary units. Positive MS full-scan spectra were acquired in the *m*/*z* range 400–1500 with 70 k of resolution using an automatic gain control (AGC) target of 3 × 10^6^ and an injection time of 100 ms. Tandem MS Full-MS/ddMS^2^ are conducted on the top 8 ions with NCE fixed at 30 with a 17.5 k resolution, AGC of 1e^5^, IT fill time of 100 ms, and isolation window of 4 *m*/*z*. The Full-MS/ddMS^2^ raw files were processed by using ProteomeDiscoverer^®^ (version 2.4, Thermo Fisher Scientific) software with the following conditions: *A. tumefaciens* as database (Uniprot, Hinxton, UK), trypsin as enzyme with max. 2 missed cleavages, min. and max. length of peptides equal to 6 and 144 amino acids, respectively, 10 ppm and 0.02 Da as tolerance for precursor and fragment ions, respectively, metoxidation, acetyl, met-loss, and met-loss + acetyl as dynamic modifications, and carbamidomethylation of cysteines as a static modification.

The main electrospray and ion optic parameters adopted for Velos Pro were the following: sheath gas flow rate, 35 (arbitrary units); auxiliary gas flow rate, 5 (arbitrary units); spray voltage, 3.5 kV; capillary temperature, 320 °C; S-lens radio frequency level, 60 arbitrary units. Positive MS full-scan spectra were acquired in the *m*/*z* range 200–2000, while MS/MS experiments based on low-energy CID were executed at collision energy 35% (a 400% value corresponds to a 100 V excitation voltage) using a 1 *m*/*z* unit wide isolation window centred on the monoisotopic *m*/*z* value. The control of the LC-MS instrumentation and the first processing of data were achieved by the Xcalibur software 2.2 SP1.48 (Thermo Scientific). The post analyses data were processed by using SigmaPlot 11.0 to graph final mass spectra for both MALDI-MS and RPLC-ESI-MS.

## 5. Conclusions

While numerous studies have focused on highlighting the potential of phages in controlling *A. tumefaciens*, there is a lack of research specifically investigating the molecular costs or fitness impacts associated with phage resistance in this bacterium. Understanding these aspects is essential for developing sustainable and effective phage-based biocontrol strategies and leveraging the bacterial costs of phage resistance. In this study, we focused on developing a sustainable strategy that combines phages with AMPs to combat *A. tumefaciens* infections. We investigated the consequences of phage resistance development in *A. tumefaciens* and demonstrated the importance of combining phages with other antimicrobials to harness the indirect effects of phages on bacterial fitness, thereby enhancing disease control. However, further research is needed to explore the molecular mechanisms of *A. tumefaciens* resistance to PAT1 infection, including genomic and transcriptomic analyses, and whether the phage-resistant mutants pay comparable fitness costs. To advance its practical use, future research should prioritize formulation optimization, rigorous evaluation under field conditions, and exploration of synergistic effects with additional antimicrobial peptides or biological control agents. These measures will be essential to ensure efficacy, stability, and sustainability while reducing dependence on chemical bactericides.

## Figures and Tables

**Table 1:** List of protein families identified in wild-type and/or AT-M1 mutant samples after SDS-PAGE separation and peptide sequences with corresponding *m*/*z* and related bands.

Protein Families	Peptides	*m*/*z*	Band	Log2 Fold Change WT/ATM1
Aspartate ammonia-lyase	LIESAALLHEINLGATAIGTGLNAPR	872.484^3+^	A-A′	0.76
	AVENFQITGVTIGHNPYLVR	743.403^3+^		0.56
	GYLTEEQLQQALSPR	866.951^2+^		0.49
	TFVVMLGEDQAR	683.349^2+^		0.59
	TQLQDAVPMTLGQEFR	917.463^2+^		0.18
ATP synthase subunit beta	FTQAGSEVSALLGR	718.385^2+^	A-A′	−0.36
	MLDPMIVGEEHYEVAR	630.306^3+^		−0.53
	TIAMDSTEGLVR	646.832^2+^		−0.52
	IMNVIGEPVDEAGPIVTAK	977.025^2+^		−0.24
Chaperonin GroEL	AAVQEGIVPGGGVALLR	803.969^2+^	A-A′	−0.09
	QIGLDIAEAMQR	672.854^2+^		−0.95
	TNDIAGDGTTTATVLAQAIVR	1044.555^2+^		−0.38
Elongation factor	LLDQGQAGDNIGALVR	820.439^2+^	A-A′ B-B′ C-C′	0.20
	VDQVDDAELLELVELEVR	1042.547^2+^		1.28
	VVIATEDNSYVER	747.879^2+^		0.13
ABC transporter substrate-binding protein	IDDLIFAITPDAAVR	815.452^2+^	A-A′	0.57
	DFNADDVIFSYNR	788.362^2+^		0.73
Flagellin	SIGNNMETTQGR	654.308^2+^	B-B′	0.74
	VGSASDNAAYWSIATTMR	950.949^2+^		0.94
	QSVSNLDISDLSIYK	841.434^2+^		0.72
	ASILTNASSMAALQTLR	874.473^2+^		0.20
	SDASALSTVSDALGIGAAK	867.447^2+^		1.04
	ALQTQQQLAIQALSIANSDSQNILSLFR	1024.560^3+^		0.37
Glyceraldehyde−3-phosphate dehydrogenase	GILGYTEEPLVSR	717.389^2+^	B-B′	1.12
	VLSWYDNEWGFSNR	886.904^2+^		0.05
Ribosomal subunit protein	VATVVAAPASQLAR	677.400^2+^	A-A′ C-C′	−0.74
	IENALGEAVLSR	636.351^2+^		−1.22
	LLGLLNAPATR	569.854^2+^		−1.54
	SAGETGQLYGSVAAR	733.865^2+^		−1.46
	TLPEFSPGDTLR	666.843^2+^		0.82
	SLATLPSLDELR	657.866^2+^		1.75
Oxidoreductase	ATGNYEQALADFIAR	820.407^2+^	C-C′	2.26
	TVVITAAGQGIGR	621.865^2+^		1.96
Superoxide dismutase	AFELPELPYDYDALAPYMSR	1181.066^2+^	C-C’	2.01
	MAFELPELPYDYDALAPYMSR	1246.579^2+^		1.18
DNA-binding protein	NPSTGAEVDIPAR	663.836^2+^	D	>10
	LAGFGSFSVSR	564.295^2+^		>10
	GRNPSTGAEVDIPAR	770.397^2+^		>10
	MNKNELVSAVAEK	716.877^2+^		>10

**Table 2:** List of antimicrobial peptides used against *A. tumefaciens*.

Peptide	Sequence	Origin	References
Ascaphin-8	GFKDLLKGAAKALVKTVLF-NH2	Frog	[37]
Lycotoxin I	IWLTALKFLGKHAAKHLAKQQLSKL	Spider	
Maculatin 1.3	GLLGLLGSVVSHVVPAIVGHF-NH2	Frog	
Piscidin 1	FFHHIFRGIVHVGKTIHRLVTG	Fish	

## Data Availability

Data is contained within the article or Supplementary Material.

## Supplementary Materials

The following supporting information can be downloaded at: https://www.mdpi.com/article/10.3390/ijms26199355/s1.
