# Supplementary material for: Synergistic Biocontrol of Agrobacterium tumefaciens by Phage PAT1 and Ascaphin-8: Enhanced Antimicrobial Activity and Virulence Attenuation via HupB Loss
Source: Int J Mol Sci. 2025 Sep 25;26(19):9355. doi: 10.3390/ijms26199355 (PMC12524860; doi:10.3390/ijms26199355)
Supplement: Supplementary file 1 [file ijms-26-09355-s001.zip › ijms-3865599-supplementary.pdf]

Supplementary material

Synergistic Biocontrol of *Agrobacterium tumefaciens* by Phage PAT1 and Ascaphin-8: Enhanced Antimicrobial Activity and Virulence Attenuation via HupB Loss

Miloud Sabri [1 #], Kaoutar El Handi [1 #], Cosima Damiana Calvano[* 2], Mariachiara Bianco [2], Angelo De Stradis [3], and Toufic Elbeaino [1,4*]

[1] International Centre for Advanced Mediterranean Agronomic Studies (CIHEAM of Bari), Via Ceglie 9, 70010 Valenzano (Ba), Italy.

[2] Interdepartmental SMART Center, Department of Chemistry, University of Bari, Via E. Orabona 4, 70126 Bari, Italy.

[3] National Research Council of Italy (CNR), Institute for Sustainable Plant Protection (IPSP), University of Bari, Via Amendola 165/A, 70126 Bari, Italy.

[4] National Research Council of Italy (CNR), Institute for Sustainable Plant Protection (IPSP), Piazzale Enrico Fermi, 1- 80055 Portici (NA), Italy.

***Correspondence:** Toufic Elbeaino (elbeaino@iamb.it); Cosima Damiana Calvano (cosimadamiana.calvano@uniba.it)

[#] These authors share first authorship.

Supplementary Table S1 List of proteins retrieved from PMF and coverage in % in wild type or/and mutant (proteins with at least 10% of coverage are considered).

Protein name	WT Cov %	AT-M1 Cov %
DNA ligase	14.8	14.4
Malate synthase G	17.3	14.1
Cysteine--tRNA ligase	18.8	13.2
Threonine--tRNA ligase	21.6	15.1
Transaldolase	26.5	26.5
Elongation factor Ts	14.6	12.3
Peptide chain release factor 1	23.7	21.4
Putative aminoacrylate hydrolase RutD	12.2	12.2
Protein RecA	18.3	--
Tetraacyldisaccharide 4'-kinase	14.4	11
50S ribosomal protein L11	25.9	--
Dihydroxy-acid dehydratase	16	14.5
UDP-N-acetylenolpyruvoylglucosamine reductase	18.8	12.3
Transcription elongation factor GreA	26.6	33.5
ATP-dependent protease ATPase subunit HslU	18.1	15.1
50S ribosomal protein L32	36.1	--
Single-strand DNA-binding protein	17.8	15
Opine oxidase subunit A	10	10
Integration host factor subunit beta	37.3	37.3
50S ribosomal protein L2	21.6	21.6
NH(3)-dependent NAD(+) synthetase	12	--
50S ribosomal protein L1	28	20.7
ATP synthase gamma chain	18.4	17.3
Ribonuclease 3	23	24.3
GMP synthase	11	10
Nicotinate phosphoribosyltransferase	10	--
DNA replication and repair protein RecF	15.7	--
Sugar fermentation stimulation protein	15.7	--
30S ribosomal protein S18	54.9	--
Transcriptional repressor NrdR	27.8	--
Pterin deaminase	16.7	--
Peptidyl-tRNA hydrolase	20.6	--
Pyrroloquinoline-quinone synthase	20.6	17
Wide host range VirA protein	--	--
Adenylate dimethylallyltransferase	--	12.3

Chaperone protein DnaJ	--	22.7
Protein GrpE	--	11.8
Conjugal transfer protein TraA	--	17.6
50S ribosomal protein L16	--	32.1
30S ribosomal protein S13	--	25.4
ATP-binding protein ChvD	--	16.4
Polyribonucleotide nucleotidyltransferase	--	17.2
Urease subunit gamma	--	22
Acetyl-coenzyme A carboxylase carboxyl transferase subunit beta	--	11.9
ATP-dependent protease subunit HslV	--	18.9
30S ribosomal protein S3	--	21.6
30S ribosomal protein S6	--	26.7

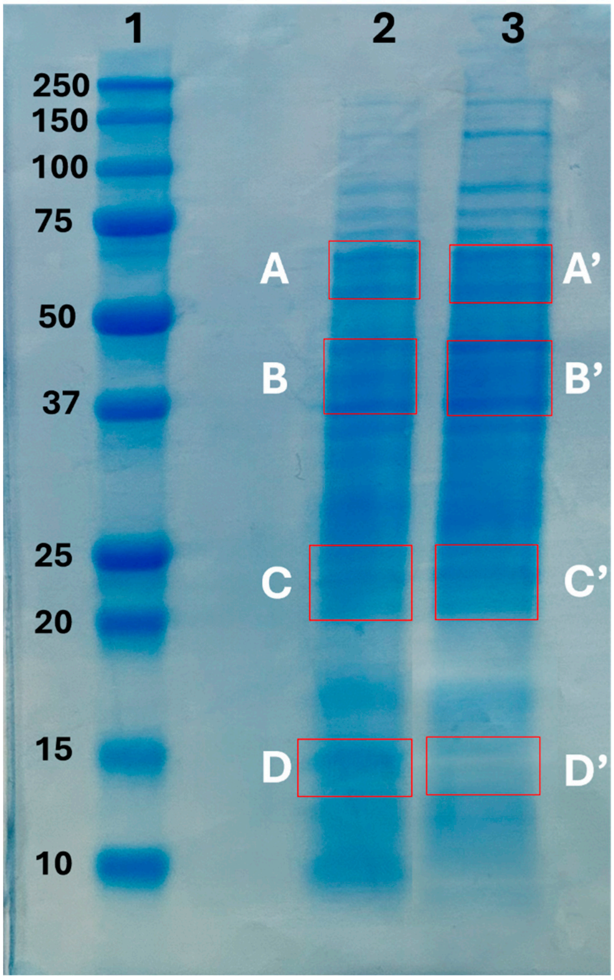

Supplementary Figure S1 SDS-PAGE gel of protein extract. Lane 1: Prestained Protein Standards, lane 2: wild type, and lane 3: mutant AT-M1.

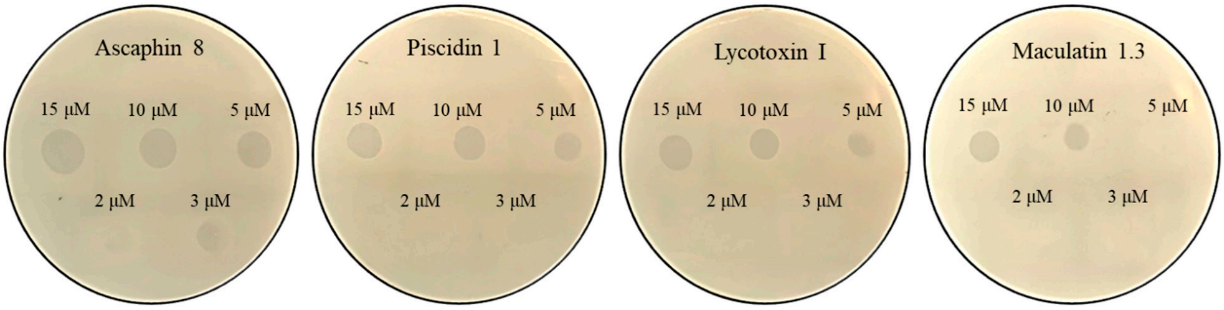

Supplementary Figure S2 YPGA agar plate showing spot assays of Ascaphin 8, Lycotoxin I, Piscidin 1, and Maculatin 1.3 at various concentrations against *A. tumefaciens* CFBP 5770.
